# Nontraumatic Spinal Cord Injury: Epidemiology, Etiology and Management

**DOI:** 10.3390/jpm12111872

**Published:** 2022-11-08

**Authors:** Diana M. Molinares, David R. Gater, Scott Daniel, Nicole L. Pontee

**Affiliations:** 1 Department of Physical Medicine and Rehabilitation, University of Miami Miller School of Medicine, 1611 1095 NW 14th Terrace, Miami, FL 33136, USA; 2 Christine E. Lynn Rehabilitation Center for the Miami Project to Cure Paralysis, Miami, FL 33136, USA; 3 The Miami Project to Cure Paralysis, University of Miami Miller School of Medicine, Miami, FL 33136, USA

**Keywords:** nontraumatic spinal cord injury, tetraplegia, paraplegia, autonomic dysfunction, neurogenic bladder, neurogenic bowel

## Abstract

The spinal cord is a conduit within the central nervous system (CNS) that provides ongoing communication between the brain and the rest of the body, conveying complex sensory and motor information necessary for safety, movement, reflexes, and optimization of autonomic function. After a traumatic spinal cord injury (SCI), supraspinal influences on the peripheral nervous system and autonomic nervous system (ANS) are disrupted, leading to spastic paralysis, sympathetic blunting, and parasympathetic dominance, resulting in cardiac dysrhythmias, systemic hypotension, bronchoconstriction, copious respiratory secretions, and uncontrolled bowel, bladder, and sexual dysfunction. This article outlines the pathophysiology of the less reported nontraumatic SCI (NTSCI), its classification, its influence on sensory/motor function, and introduces the probable comorbidities associated with SCI that will be discussed in more detail in the accompanying manuscripts of this special issue. Finally, management strategies for NTSCI will be provided.

## 1. Epidemiology

There are currently 299,000 individuals in the USA with traumatic spinal cord injury (TSCI), with an annual incidence of 54 cases per 1 million people, i.e., approximately 18,000 new cases per year [1,2,3]. Additionally, prevalence data for non-traumatic SCI (NTSCI) in Canada indicates 1120 per million population [4]; extrapolation to the USA would yield another 372,000 persons with NTSCI. In several countries, the incidence of NTSCI has been reported as higher than TSCI, and it is expected to continue to increase with the aging of the population [5,6]. Although there are distinct documentation and databases where TSCI are reported, there are fewer studies and publications on NTSCI. In 2013, New et al. published a global map where the epidemiologic characteristics of NTSCIs were highlighted [6]. High-income countries, including those in North America, tend to find a higher proportion of cases secondary to degenerative conditions (54%) and tumors (26%), while in developing countries, there is a higher number of infections such as HIV and tuberculosis. The authors also highlighted the proportionally decreased access to SCI rehabilitation centers for NTSCI patients compared to TSCI patients, although this could be secondary to under reporting [6,7,8]. Contrarily, in other studies, NTSCI represents 60% of spinal cord injury admissions to the rehabilitation unit [9]. Even though there is a lack of epidemiological studies, the International Spinal Cord Society reported a calculated NTSCI incidence of 6 to 76 new cases per million per year [10].

NTSCI causes are very variable and heterogeneous, and recent international guidelines have been developed to classify them in a systematic fashion [6]. Axis 1 in this nomenclature is to distinguish those NTSCI that are congenital (i.e., spinal dysraphism, Chiari malformations, and skeletal malformations) or genetic disorders (hereditary spastic paraparesis, spino-cerebellar ataxias, adreno-myeloneuropathy, leukodystrophies, and spinal muscular atrophy), as opposed to those that are acquired (vertebral column degenerative disorders, metabolic disorders, vascular disorders, inflammatory/autoimmune diseases, radiation-related, toxic, neoplastic, infectious, and miscellaneous) [6]. Acquired NTSCI is usually associated with older age individuals, with degenerative spinal column conditions being the most common causes. Other common acquired conditions include benign or malignant tumors, vascular problems, infectious or inflammatory processes [5,11]. NTSCI has been most often characterized as having incomplete lesions, and is most often associated with paraplegia. A cohort study of 112 subjects, conducted in Italy, corroborated reports of demographic characteristics of NTSCI, including an increased incidence of these lesions in older and female patients (Table 1) [12]. The pathophysiology of the NTSCI varies based on the etiology but shares similar characteristics as those of traumatic etiology, except for the high-energy primary injury [13]. Pathogenic cascades include neuroinflammation, demyelination, apoptosis, necrosis, protein misfolding, and autophagy. The differences in the pathophysiological processes of NTSCI increase the complexity of the medical management and rehabilitation process. Additionally, these patients often have pre-existing medical comorbidities, including diabetes, cardiovascular and pulmonary disease, as well as dementia at the time of injury. Older age and cognitive deficits are associated with poor carryover of rehabilitation interventions, while medical comorbidities decrease participation and efficiency [14].

## 2. Etiologies of Nontraumatic Sci

### 2.1. Congenital/Genetic Disorders

#### Congenital

Congenital etiologies of NTSCI can include spinal dysraphism, Chiari malformations, skeletal malformations, and congenital syringomyelia [6]. Spinal dysraphism is a birth defect that affects the spine due to a neural tube defect (NTD) with incomplete closure and can occur anywhere along the spine if the neural tube does not close all the way. When this occurs, the vertebra that protects the spinal cord does not form and close properly, resulting in damage to the spinal cord and nerves [5]. Spina bifida (SB) occulta is the mildest type in which there is no NTD but rather an abnormal formation of the posterior vertebra. In SB occulta, the spinal cord and nerves are unaffected, and there is typically no significant disability. Meningocele is the moderate form of SB in which a sac of fluid protrudes through an opening in the neural tube but does not contain the spinal cord or nerves, such that there is usually little or no neurologic damage and unlikely disability. The most severe but most common type of SB is the myelomeningocele, which is among the most complex congenital anomalies compatible with life [15]. With this entity, the sac of fluid protruding through the opening of the vertebral defect contains portions of the spinal cord and nerves. Myelomeningocele subsequently results in various disabilities depending on the level and extent of the defect. SB is one of the most common childhood disabilities [16], and has been associated with increased mortality and disability requiring lifelong medical care [17,18]. Each year, about 1427 babies are born with SB in the United States, or 1 in every 2758 live births [19], although the incidence has decreased over the last several decades.

Chiari malformations are often associated with SB and are progressively classified into (1) Type 1, abnormal extension of the cerebellar tonsils below the foramen magnum; (2) Type 2, additionally displaced medulla and 4th ventricle; (3) Type 3, additionally displaced cerebellar and brainstem tissue extending into an infra-tentorial meningocephalocoele; and (4) Type 4, cerebellar and brainstem hypoplasia [6]. Of note, while the malformations are static, symptoms can worsen as the child grows if the spinal cord is tethered and stretched; frequent surveillance is indicated, particularly if there are changes in motor or sensory function [18].

Congenital syringomyelia most often occurs coincident with SB or Chiari malformations, but may be idiopathic [20]. If found incidentally and the patient is asymptomatic, conservative management/surveillance is recommended. However, if ascending sensory and/or motor loss occurs, especially in the presence of new bladder/bowel incontinence, surgical intervention/shunting is warranted [18,20].

### 2.2. Genetic Disorders

#### 2.2.1. Hereditary Spastic Paraplegia (HSP)

HSP encompasses a diverse group of genetic disorders. There are over 70 genetic types of HSP identified, with SPG3A, SPG4, and SPG11 as the most common and well described [21]. HSP is frequently inherited in an autosomal dominant pattern, with autosomal recessive patterns appearing less commonly. The onset of symptoms is typically insidious and varies from infancy to adulthood. HSP is associated with axon degeneration involving the lateral corticospinal tracts and the fasciculus gracilis tract, with secondary demyelination [21]. Clinical hyperreflexia, lower extremity weakness, and spastic gait are observed across all genotypes. Bladder and bowel incontinence and a vibratory sensation deficit may be observed [21]. Some types of HSP may be associated with intellectual disability. Genetic testing panels of HSP may permit confirmation of diagnosis in most cases, though HSP remains largely a clinical diagnosis. No current treatments are available for HSP, but management centers around spasticity and rehabilitative management [21].

#### 2.2.2. Friedreich’s Ataxia

Friedreich’s ataxia, a neurodegenerative condition secondary to the FRDA gene mutation that causes reduced levels of frataxin, the mitochondrial protein involved in iron regulation. It is the most inherited ataxia, occurring in about 1 in 50,000 individuals with a carrier prevalence estimated to be 1 in 110. It is inherited in an autosomal recessive pattern [22]. Dorsal root ganglia, posterior columns of the spinal cord, and corticospinal tracts are most affected by this mutation. Patients present generally prior to age 25 with progressive and unremitting ataxia with loss of deep tendon reflexes [22]. Secondary features include dysarthria and extensor plantar responses. Cardiac involvement primarily takes the form of hypertrophic cardiomyopathy and, less commonly, hypokinetic cardiomyopathy with some evidence of cardiac iron deposition. Electrodiagnostic studies typically demonstrate absent sensory nerve action potentials and reduced motor nerve conduction velocities [22]. Management of the disease focuses on rehabilitative functional goals and possible spinal corrective surgery for severe cases of scoliosis [23].

#### 2.2.3. Spinal Muscular Atrophy

Spinal muscular atrophy (SMA) is an autosomal recessive disease resulting from deletion of the SMN1 gene in the vast majority of cases. It causes the loss of function of the survival motor neuron (SMN) protein, which is present in all cells and is involved in pre-mRNA splicing. SMA is divided into subtypes: SMA type 1 (Werdnig–Hoffmann disease), SMA type 2, and SMA type 3 (Kugelberg–Welander syndrome). The severity of the disease lessens with the progression of the types, with SMA type 1 characterized by hypotonia and inability to sit and a life expectancy of less than two years. Patients with SMA type 2 progress more slowly and do not develop the ability to walk. Those with SMA type 3, the mildest form, lose the ability to walk after gaining the function and have relatively normal life expectancies [24]. Loss of SMN correlates with atrophy and loss of motor neurons with astrocytosis in the spinal anterior horns and brain stem motor nuclei, resulting in evidence of denervation at the level of the muscle. More recently, antisense oligonucleotide therapeutic agent nusinersen gained FDA approval in 2016 for the use of altering splicing of SMN2 pre-mRNA to increase the amount of functioning SMN protein [24].

### 2.3. Acquired Ntsci

#### Degenerative Spine Disease

Cervical spondylitic myelopathy is the most common non-traumatic progressive spinal cord disorder [25]. The prevalence is around 2% and is caused by spinal cord compression due to degenerative changes in the spinal column components [10]. Spinal degenerative changes include spondylosis, disc disease, ligament hypertrophy, and ossification of the ligament. While some patients can remain asymptomatic, others present with heterogenous symptoms, including bowel/bladder dysfunction, balance disturbance, and muscle weakness. Diagnostic tools include medical history, a full neurological examination, and obtaining appropriate imaging. Magnetic resonance imaging (MRI) is the most accurate imaging for the evaluation of compression of the cord secondary to degenerative spinal conditions [25]. A meta-analysis that included 3786 subjects evaluated the prevalence of asymptomatic and symptomatic spinal cord compression on MRI and found spinal cord compression in 20% of asymptomatic and 86% of symptomatic individuals [10]. Surgical decompression is recommended for moderate to severe cases as well as those with progressive symptoms. Unfortunately, some cases are underdiagnosed and not referred to surgical teams at an appropriate time. Delays in surgical decompression can lead to poor neurological and functional outcomes [25].

### 2.4. Metabolic

#### Subacute Combined Degeneration (Vitamin B12 Deficiency)

Vitamin B12 is required in the process of myelination of the central nervous system. Thus, with vitamin B12 deficiency, demyelination of the brain and spinal cord, particularly in the areas of the cervical and thoracic dorsal and lateral columns, and peripheral nerves, may occur, which is known as subacute combined degeneration (SCD). Demyelination can occur in a patchy, “spongiform” demyelination of the spinal cord with axonal degeneration; foamy macrophage permeation and reactive astrocytosis contribute to the overall degeneration [26,27]. Deficiency of B12 may arise from pernicious anemia, dietary deficiency, bariatric intervention, toxic exposure to nitrous oxide, and medication use related to reduced gastric acid production [28,29]. Clinically, patients present with macrocytic anemia and progressive sensory loss, namely affecting proprioception and vibratory sensation, diminished deep tendon reflexes, and later developing motor weakness and spastic ataxic gait. Bowel and bladder incontinence and sexual dysfunction may also develop. Neurological sequalae are often responsive to high-dose cobalamin supplementation if treated promptly [30].

### 2.5. Vascular Disorders

#### 2.5.1. Hemorrhage

Vascular hematomas can cause compression of the spinal cord. Patients usually present with back pain and neurological deficits characterized by weakness, as well as radicular pain symptoms, depending on the location [31]. Bleeding can happen spontaneously in patients with underlying anticoagulation, antiplatelet medications, or those with coagulopathies. Hematoma evacuation is necessary in most cases, especially when associated with neurological deficits [31].

#### 2.5.2. Vascular Malformations

Arteriovenous malformations can lead to ischemic or hemorrhagic interruption of circulation via congestive myelopathy or hemorrhage, with nidus types being associated with hemorrhage, and fistulous types associated with congestive myelopathy [32].

#### 2.5.3. Ischemia

Spinal cord infarction occurs from vascular compromise of the anterior spinal artery or its preceding vessels. Ischemia of the spinal cord can be secondary to thrombotic, embolic, vaso-occlusive, or hemorrhagic etiologies. Disruption in flow from arteriovenous malformation or aortic aneurysm or dissection can also lead to infarction. The anterior spinal artery is supplied rostrally by branches off the vertebral artery and distally from the artery of Adamkiewicz. Due to the anatomical distribution of segmental arteries to the spinal cord and the lack of redundancy supplying the anterior two thirds, compromise to significant portions of the thoracic spinal cord can be achieved through single vessel occlusion. Patients may initially present with back pain due to preservation of dorsal columns, and flaccid paraplegia below the level of the lesion [33].

### 2.6. Inflammatory/Autoimmune Diseases

#### 2.6.1. Acute Transverse Myelitis

Acute Transverse Myelitis (ATM) is a fairly rare neurological condition that results in approximately 1.3–4.6 cases per million per year. The etiology of ATM may be post-infectious (related to cytomegalovirus, herpes simplex type 1 or 2, varicella-zoster or Epstein–Barr virus, and more recently associated with Zika virus [34] and SARS-CoV2, [35]), drug or toxin-related, or paraneoplastic [36]. ATM has been linked to immunological response, as supported by temporal association with vaccination [37]. Other etiologies include systemic autoimmune disorders, systemic lupus erythematosus or sarcoidosis, and acquired demyelinating diseases such as multiple sclerosis [36]. ATM may also be idiopathic, but alternate diagnoses must be ruled out. For an idiopathic diagnosis to be made, the patient must demonstrate bilateral sensory, motor, or autonomic dysfunction attributed to the SCI with a clearly defined sensory level, bilateral signs or symptoms, lack other etiology of SCI or infectious/autoimmune etiology, and show inflammation in the spinal cord via cerebral spinal fluid and a progression of symptoms between 4 h and 21 days after the onset of symptoms [38]. In all cases of ATM, cerebral spinal fluid analysis may yield lymphocytic pleocytosis and elevated protein. Many cases are characterized by perivascular infiltration, demyelination, and monocytic and lymphocytic axonal injury. Incomplete lesions have a higher tendency to progress to multiple sclerosis as compared to complete ATM-associated lesions [39]. Approximately a third of patients recover completely, a third improve with persistent neurologic deficits, and a third do not improve at all [40].

#### 2.6.2. Acquired Demyelinating Disorders

Multiple sclerosis (MS) is an autoimmune-mediated disorder characterized by CNS lesions that affect the white matter. The pathogenesis involves focal T-lymphocytic and macrophagic attack on the myelin sheath, resulting in CNS plaque formation [41]. Depending on the location of the extent of the lesions, patients can present with sensory and motor deficits, spasticity, or ataxia, as well as bowel and bladder dysfunction associated with spinal cord involvement. Classical findings, such as Lhermitte’s sign, a descending electrical sensation along the spine in response to neck flexion, and Uhthoff’s phenomenon, relative heat sensitivity with neurologic worsening, are indicative of an MS diagnosis. The 2021 MAGNIMS-CMSC-NAIMS international consensus recommends the use of MRI in the diagnosis of and prognostication of disease burden in MS with standardized brain and spinal cord protocols, with a focus on three-dimensional acquisition techniques for diagnosis and monitoring [42]. Subtype identification assists in prognostication of functional and neurological outcomes. Subtypes include relapsing remitting MS (RRMS), primary progressive MS (PPMS), secondary progressive MS (SPMS), and progressive relapsing MS (PRMS) [41,43]. Management of the disease focuses on control of acute flares with steroids, disease modification, and immune modulation. Symptomatic treatment is achieved with fatigue, depression, and spasticity management.

Neuromyelitis optica spectrum disorders (NMOSD) are distinct CNS inflammatory diseases that, while less common than MS, can significantly affect the spinal cord. The discovery of water channel aquaporin-4 (AQP4) IgG and its associated pathogenicity permits further categorization of the disorders [44]. In 2015, core clinical features were determined by international consensus and include (1) optic neuritis, (2) acute myelitis, (3) area postrema syndrome, (4) acute brainstem syndrome, (5) symptomatic narcolepsy, and (6) symptomatic cerebral syndrome. Diagnostic criteria for NMOSD with AQP4-IgG require at least one core clinical characteristic with a positive AQP4-IgG test. Diagnosis for NMOSD without AQP4-IgG is met with two core clinical characteristics, one of them being optic neuritis, acute myelitis with longitudinally extensive transverse myelitis lesions, or area postrema syndrome with dissemination in space as well as the absence of a positive AQP4-IgG test [45].

#### 2.6.3. Neoplasms

Primary spinal cord tumors are less common than secondary spinal tumors that compress the spinal cord. Primary tumors can be in the intramedullary, i.e., within the substance of the cord, or intradural, i.e., between the cord and dura, space. Intramedullary tumors account for approximately 4–5% of all primary central nervous system lesions. The majority of intramedullary tumors (56%) are benign, with ependymomas (60%) and astrocytoma (30%) being the most common diagnoses [46]. Symptoms depend on the location of the tumor, however, in most cases, patients present with acute or subacute onset of neurological deficits without pain.

Extramedullary intradural tumors are mostly represented by meningiomas (33%) and nerve sheet tumors (27%) (Table 2) [46]. Symptoms are usually localized depending on the location of the tumor (Table 3). They can present as different spinal cord syndromes, such as Brown–Sequard syndrome. However, the clinical presentation generally includes bilateral motor and sensory signs and symptoms.

## 3. Benign Neoplasms

**Figure 1:**
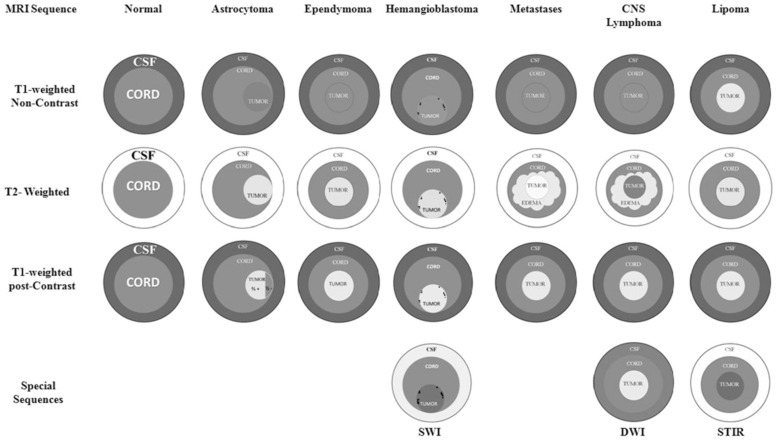
MRI appearance of different intramedullary spinal tumors. Adapted with permission from reference [48] Mechtler, L.L.; Nandigam, K. Spinal cord tumors: new views and future directions. *Neurol. Clin.* **2013**, *31*, 241–268.

Ependymoma, the most common intramedullary tumor, is more predominant among males, with a mean age of presentation of around 40 years [47]. The majority (67%) of the tumors arise from the cervical spinal cord, and around half of the cases are associated with the presence of syringomyelia [46]. Most ependymomas are benign, however, a minority are classified as anaplastic ependymomas, which have malignant behavior with progression [47]. MRI is the preferred imaging modality for the diagnosis of ependymoma and other spinal cord tumors [10]. Although other imaging techniques, including PET scan and CT mylography, can provide information in regards of the presence of occupying mass in the spinal cord, but are unable to evaluate the characteristics of the tumors. MRI provides superior resolution, the most precise locations, and the ability to evaluate therapeutic results [48]. The characteristics of the tumor in the T1, T2, and STIR images as well as the use of gadolinium during MRI study can help guide the histological diagnosis and treatment approach [10,48]. Ependymomas are centrally located and enhance with the use of contrast, versus astrocytomas that are usually found in the periphery of the spinal cord and only 2/3 of tumors enhance with the use of contrast (Figure 1) [48].

Surgical resection when possible is the mainstem treatment of spinal cord tumors. Neurological and functional outcomes are driven by pre-operative neurological deficits as well as pathological grading of the tumor. Total gross surgical resection is not always achieved; in those cases, depending on the grading of the tumor, further treatment may be recommended. For low-grade tumors, surveillance with serial MRIs is recommended, while for high-grade tumors, stereotactic radiation therapy is often required [48]. Spinal lesions tend to be associated with sphincter disturbances only with extensive bilateral damage (Table 3).

## 4. Malignant Neoplasms

Malignant primary spinal cord tumors include glioblastomas and high-grade astrocytomas [6].

Secondary malignancies are the most common tumors of the spinal column with a reported prevalence of 70% of patients with cancer; ten percent of patients with spine metastasis (from breast, lung, prostate, and multiple myeloma) develop spinal cord injuries due to tumor compression [49]. The most common injuries arise from extradural masses causing epidural cord compression. Similarly, fractures of the vertebral bodies from lytic or blastic bony lesions can result in retropulsion of bone fragments with vertebral body collapse. Patients usually present with back pain, weakness, sensory deficits, and bowel/bladder dysfunction. Thirty-five to 75% of the patients have motor deficits [49,50]. Functional pain is suggestive of fracture or spinal instability and is usually worsened by movement, sneezing, coughing, and lying flat. Lesions can involve the cervical, thoracic, and lumbar spine; however, the majority of the lesions are located in the thoracic spine. A clinical presentation suggestive of spinal metastases should prompt an urgent spine MRI, especially if there is evidence of neurological deficits. MRI is the gold standard test with a sensitivity of 93% and a specificity of 97%; a CT myelogram is only recommended when MRI is contraindicated [51].

Initial treatment of neoplastic cord compression syndrome includes the use of corticosteroids to improve pain control and preserve neurological function by decreasing cord edema. Additionally, patients often require the use of opioids for the management of pain. In cases of spinal instability, surgery and radiation play an important role. Vertebroplasty and kyphoplasty are generally not indicated in the presence of neurological deficits. The Spine Instability Neoplastic Score (SINS) can help assess the need for surgical intervention (Table 4) with a sensitivity of 96% and a specificity of 79%; a score greater than 12 is indicative of spinal instability [51,52,53,54]. Radiation alone instead of post-operative radiation depends on several factors, including the patient’s prognosis, pain level, radiosensitivity of the tumor, and degree of compression. The use of the neurologic, oncologic, mechanical, and systemic disease (NOMS) decision framework can assist providers in deciding which treatment options to pursue [52].

### 4.1. Infection

#### 4.1.1. Spinal Epidural Abscesses

Spinal epidural abscesses account for 0.1–1.2 cases per 10,000 hospitalizations, with a mortality rate of 13–16% [55]. Staphylococcus aureus is the most common pathogen related to spinal epidural abscesses, with the thoracic spine being most frequently involved [56]. Patients can present with a triad of fever, back pain, and neurological deficits; however, the diagnosis can be very challenging. The patient’s medical history plays a significant role in the diagnosis of epidural abscess. Patients with a recent history of lumbar puncture, epidural anesthesia, and epidural injections are at higher risk. Similarly, patients with diabetes, cancer, a history of immunosuppressants, renal failure, and a history of intravenous drug use are more vulnerable to these types of infections. Despite obtaining history, risk factors, and physical examinations, diagnoses remain challenging, and imaging is often necessary for a complete evaluation. Compression from the mass effect of the collection can occupy several continuous or noncontinuous levels. MRI with gadolinium is the gold standard to diagnose epidural abscess, however it is difficult to visualize in some cases [57]. For treatment, surgical evacuation has been shown to have better outcomes than antibiotics alone [55].

#### 4.1.2. Viral Infections

Various viral infections can contribute to myelopathic clinical presentations of unique etiology. Herpes viruses may cause spinal abscesses, myelopathy, and subsequent SCI. Similarly, retroviruses such as human immune virus (HIV) and human T-lymphotropic virus type 1 (HTLV-1) can result in SCI. Human immunodeficiency virus (HIV)-associated myelopathy, also known as vacuolar myelopathy (VM), is characterized by spastic paraparesis in advanced HIV/AIDS that starts in the thoracic cord and progresses rostrally [58]. Patients present with lower extremity weakness in the absence of pain, with sensory and proprioceptive deficits, imbalance, and bowel/bladder incontinence. Disease pathology localizes to the posterolateral columns of the spinal cord with early microglial activation and macrophagic infiltration. This results in vacuolar formation and myelin pallor or demyelination, leading to spongiform degeneration of the spinal cord. There is evidence of axonal swelling and reactive astrocytosis. One case-control study found that VM was associated with mycobacterium avium intracellulare and pneumocystis jirovecii infection at the time of autopsy, suggesting that VM is associated with advanced disease [27]. There are no direct therapies for the treatment of VM; management of AIDS with highly active antiretroviral therapy (HAART) is the mainstay of treatment, but there is a lack of evidence of its effect in VM; some case reports have identified agents such as intravenous immunoglobulin as potential therapeutic agents for VM [58].

HTLV-1is a blood-borne pathogen endemic to areas in the Caribbean, South America, parts of Africa, and Southern Japan. HTLV-1 viral infection may result in an asymptomatic carrier state, adult T-cell leukemia lymphoma (ATLL), or HTLV-1 associated myelopathy/tropical spastic paraparesis (HAM/TSP). HAM/TSP affects adults aged 40–50 years, with a higher incidence in women compared to men. It may present insidiously with gait abnormalities, hyperreflexia, lower extremity weakness, lower back pain, and urinary dysfunction. HAM/TSP occurs in approximately 2–5% of patients infected with the virus, resulting from destruction of the central nervous system due to chronic inflammation from lymphocytic immune activation. Demyelination and axonal degeneration along the pyramidal tract have been noted with thoracic spinal cord atrophy [59,60]. Treatment requires on inflammation reduction with steroids and alpha-interferon; however, newer biological agents are currently under investigation.

Post-polio syndrome (PPS) is the neurological manifestation years after an initial infectious insult by poliovirus, a single-stranded RNA enterovirus. While the pathophysiology is poorly understood, it is believed that the initial viral insult results in degeneration of motor neurons with resulting muscle fiber denervation. Surviving motor neurons go on to supply a greater number of muscle fibers via axonal sprouting, electrodiagnostically manifesting as jitter and blocking [61]. When these new neural connections decompensate, new neurologic dysfunction in the form of PPS affecting the anterior horn cells is presumed. Updated diagnostic criteria based on a committee of experts define PPS with the following criteria: (1) prior paralytic poliomyelitis with evidence of motor neuron loss confirmed by history of the acute paralytic illness, signs of residual weakness and atrophy of muscles on neurologic examination, and signs of denervation on electromyography; (2) A period of partial or complete functional recovery after acute paralytic poliomyelitis, followed by an interval (usually 15 years or greater) of stable neurologic function; (3) gradual or sudden onset of progressive and persistent new muscle weakness with or without generalized fatigue, muscle atrophy, or muscle or joint pain; (4) persistence of the condition for a least one year; and (5) exclusion of other neurologic, medical, and orthopedic causes for patient findings [62]. Biomechanically, patients with PPS typically present with asymmetric limb involvement and possible accompanying limb length discrepancy, contractures, and spinal deformities [63].

#### 4.1.3. Neurosyphilis

Mycobacterium treponema pallidum is responsible for the development of syphilis. While rarely seen given improved detection and treatment of syphilis, neurosyphilis is the neurological manifestation of long-term, untreated syphilis [64,65]. Neurosyphilis is divided into early and late stages. Early stages are associated with asymptomatic meningitis and syphilitic meningomyelitis; the latter is a common manifestation of neurosyphilis involving the spinal cord, and develops approximately six years from the time of infection. It presents with upper motor neuron signs of spasticity, weakness, and hyperreflexia. Late stages of neurosyphilis are associated with tabes dorsalis, which is characterized by demyelination and axonal degeneration of the posterior columns of the spinal cord and dorsal root ganglia and dorsal spinal roots. The exact pathogenesis is not clearly defined. The onset latency of disease development is around 10 to 15 years and is associated with diminished proprioception resulting in imbalance and anesthesia. Patients may develop a positive Romberg sign with worsening spastic paraparetic gait and spasticity [64,65]. Greater levels of immunosuppression in HIV patient populations co-infected with syphilis correlate with the development of neurosyphilis. The use of highly active antiretroviral therapy (HAART) reduces the odds of disease development [66].

### 4.2. Miscellaneous

#### 4.2.1. Amyotrophic Lateral Sclerosis

Amyotrophic lateral sclerosis (ALS) is a rare degenerative motor neuron disease characterized by features of both upper motor neuron (UMN) and lower motor neuron (LMN) involvement. Pathologically, ALS generally spares gross changes to the brain but demonstrates anterior nerve root atrophy in the spinal cord with demyelination seen in the anterior and lateral columns, leading to loss of large motor neurons of the anterior horn and lower cranial motor nuclei [67]. ALS can occur as familial inheritance in an autosomal dominant pattern or may be sporadic, with more than 100 genes implicated in relation to sporadic ALS. 1 to 2 new cases of ALS per 100,000 are seen in the United States and Europe with approximately 10% representing familial cases and 90% are sporadic [68]. LMN features of ALS include muscle atrophy, fasciculations, while UMN features include hyperreflexia, spasticity, and pathologic reflexes. Bulbar involvement may begin at the onset, with a poor prognosis, or develop later in the course. Patients typically develop progressive symptoms that lead to global weakness and, ultimately, respiratory muscle weakness and failure. Electrodiagnostic criteria for ALS include denervation as evidenced by fibrillations, fasciculations, and positive sharp waves across different nerve roots, including bulbar muscles. Genetic testing can offer insight into familial cases of ALS. Riluzole improves 1-year survival by approximately 15% and improves overall survival by approximately 2 to 3 months. Management of ALS focuses on mobility assistance, respiratory care, and nutritional support. Phrenic nerve pacing can be considered in certain patients with ALS with respiratory dysfunction after evaluation of phrenic nerve patency [69].

#### 4.2.2. Primary Lateral Sclerosis

Differentiating primary lateral sclerosis (PLS) from ALS can be diagnostically challenging. PLS also presents as a slowly progressive neurodegenerative disease affecting motor neurons with corticobulbar involvement. Symptom onset, however, occurs in the 6th decade and demonstrates a male predominance, approximately 2–4:1. In comparison with patients with ALS, patients with PLS generally have a longer life expectancy on the level of a decade or more. Updated consensus-based diagnostic criteria revised in 2020 require: (1) the presence of symptoms at age 25 or beyond; (2) symptoms of progressive UMN dysfunction for at least two years; and (3) signs of UMN dysfunction in at least two of three regions: upper extremity, lower extremity, and bulbar. Diagnosis also requires the absence of (1) sensory symptoms; (2) LMN involvement; and (3) a more likely diagnosis. Probable PLS is defined by the absence of significant active LMN degeneration 2–4 years from symptom onset; definite PLS is defined by the absence of significant LMN degeneration four or more years from symptom onset. PLS occurs sporadically and genetic testing can help rule out other genetically defined UMN syndromes [70].

### 4.3. Mimics

#### 4.3.1. Neurological/Non-neurological Disorders

Various clinical syndromes lead to diagnostic confusion with spinal cord involvement due to apparent shared features. Bilateral small vessel disease of the brain can be mistaken for spinal cord involvement but may be differentiated by potential clues to its location with other neurological findings, such as dysphagia and cognitive or behavioral changes. Peripheral nervous system disorders such as Guillain–Barré syndrome can appear to behave like an acute myelopathic clinical picture but initially affect the peripheral nerve rather than the anterior horn cell and, as such, are excluded from the NTSCI nomenclature [6,71]. Metabolic conditions, such as thyrotoxicosis, periodic paralysis, and hypocalcemia, share features of weakness and/or hyperreflexia that may suggest an upper motor neuron lesion [72]. Somatic symptoms, such as weakness and fatigue, that may mimic neurological involvement, may also accompany psychological disorders such as anxiety and depression. In order to accurately distinguish these mimics, excellent history-taking and physical exam skills will assist in distinguishing these confounding differential diagnoses with true myelopathy. Evaluation should center on apparent discrepancies in objective findings, namely muscle strength testing compared to functional muscle use. Inconsistencies can be elucidated by testing a muscle group statically, as in a manual muscle test, and comparing it to functional usage, as in toe-walking. Maneuvers such as the Hoover’s sign, a weakness of hip extension that resolves when the contralateral hip flexes against resistance, can point toward non-organic weakness [73].

#### 4.3.2. Assessment and Classification of Ntsci

The International Standards for Neurological Classification of Spinal Cord Injury (ISNCSCI) were developed by the American Spinal Injury Association (ASIA) in 1992 [74], with revisions in 2011 [75] and, most recently, in 2019 [76]. Although initially developed to assess TSCI, the ISNSCI tool has recently been demonstrated to be reliable and valid in the assessment of NTSCI [77]. Additionally, the International Standards to determine remaining Autonomic Function after Spinal Cord Injury (ISAFSCI) should be completed to determine the risk of autonomic dysfunction, particularly for those who have SCI at or above T6 [78]. Such assessments should assist in determining comorbidities that might prolong the rehabilitation stay, including neurogenic restrictive/obstructive lung disease, neurogenic orthostatic hypotension, autonomic dysreflexia, neuropathic pain, spasticity, neurogenic bladder, neurogenic bowel, thermodysregulation, pressure injuries, and risk for venous thromboemolism.

#### 4.3.3. Ntsci Comorbidities

As with TSCI, comorbidities associated with NTSCI vary with the level and completeness of the SCI. Although lesions are more likely to be incomplete, people with NTSCI in the cervical and upper thoracic cord are at risk for dysphagia, neurogenic restrictive and obstructive lung disease, neurogenic bradycardia and dysrhythmias, neurogenic orthostatic hypotension (NOH), autonomic dysreflexia, adaptive cardiomyopathy, venous thromboembolism, neurogenic obesity, metabolic syndrome (including Type 2 diabetes mellitus, hypertension, dyslipidemia, and systemic inflammation), neuropathic pain, spasticity, neurogenic bladder, neurogenic bowel, immobilization hypercalcemia, heterotopic ossification, osteopenia/osteoporosis, pressure injuries, and risk of venous thromboembolism. These comorbidities must be considered for each person with NTSCI and incorporated into the rehabilitation plan with appropriate goals addressed.

#### 4.3.4. Rehabilitation Outcomes & Admission

As described previously, the causes of non-traumatic spinal cord injury (NTSCI) span across several organ systems, creating a large cast of culprits and subsequently providing specially designed rehabilitation units the opportunity to provide care to an ever growing and in-need patient population. Although the need may seem clearly evident from the physiatrist’s side of the veil, shedding light on the benefits of admission and outcome from treatment at a rehabilitation unit is required to help push forward adequate care guidelines. Comorbidities need to be carefully documented with detailed plans for rehabilitative care goals. New et al. performed a prospective cohort study comparing spinal cord injury patients treated at specialized spinal cord rehabilitation units versus non-specialized rehabilitation units. NTSCI patients were admitted less often to the specialized SCI care units, but those who were had greater functional outcomes [8]. It comes as no surprise that a patient with a spinal cord injury from traumatic or non-traumatic etiology benefits significantly from treatment in acute inpatient rehabilitation, but a deeper dive into the data is necessary before any significant changes can begin to materialize.

Several researchers have attempted to parse out the relevant and meaningful data with regard to the level of functional gain that can be achieved with appropriate specialized care. ASIA impairment scale (AIS), functional independence measure (FIM), Barthel index (BI), estimated length of stay, and needs assessment checklist outcomes have all been utilized to make a compelling evidence-based case for improvements in the standards of care in NTSCI patients [5,6]. Scivoletti et al. demonstrated in a study of 144 traumatic spinal cord injury patients (TSCI) and 236 NTSCI patients that after adjusting for confounders, such as age, sex, and lesion level; each patient population showed similar results in neurological and functional improvement based on BI measurements [9]. Alito et al. demonstrated in evaluation of a 112 patient cohort made up of 85 NTSCI and 27 TSCI patients, that although admission FIM scores were lower for TSCI patients at the time of discharge, both groups demonstrated nearly equivalent FIM and BI scores [12]. McKinley et al. presented a slightly different outcome but in mild opposition, demonstrating that groups of 87 NTSCI and 87 TSCI patients showed significant FIM improvements from admission until discharge, with the TSCI group having a higher discharge FIM, but the NTSCI group having a significantly shorter length of stay [79]. As with McKinley’s group, a Norwegian study by Gedde et al. also demonstrated a significant length of stay difference between NTSCI and TSCI groups, with an average of 3.4 weeks in variation [80]. Utilizing the needs assessment checklist, Kennedy and Chessell were able to provide evidence in favor of the NTSCI group for greater increases in activities of daily living, bowel management, community preparedness, and effective use of wheelchairs and equipment [81]. One significant endpoint that remained consistent between NTSCI and TSCI groups throughout multiple studies was that functional improvements were greater in incomplete versus complete spinal cord injuries.

Below in Table 5 is a summary of studies primarily looking at two highly evaluated measures of rehabilitation care, FIM and LOS. The length of stay is highly variable across countries, but consistently lower in the NTSCI group. Potential reasons to explain this discrepancy include urgency to treat spinal cord tumors with chemotherapeutic agents or radiotherapy, reduced likelihood for spine stabilizing orthotics, and less pain in those with NTSCI. Functional outcome measure tends to show a different trend in that the discharge FIM scores for both TSCI and NTSCI groups are largely similar and statistically insignificant from study to study, while admission FIM is lower in the TSCI patients. This difference in admission FIM in TSCI comes from the consistency with which those injuries result in a more severe ASIA impairment scale on presentation [82].

Rehabilitation outcomes at the moment of discharge have been studied in depth, as indicated by some of the information provided above. Several other studies have expanded on the same outcome measures with similar and consistent results. NTSCI patients undoubtedly benefit to equivalent levels at discharge as their TSCI counterparts, generally in much less time, as evidenced by shortened LOS. Currently, the field of SCI post-rehabilitation long term outcomes has a paucity of data about the retention of functional gain following rehabilitation. The majority of the data collected to date about the years following injury and acute rehabilitation is focused on comorbid conditions and sequelae that cause rehospitalization, such as: pneumonia, pressure wounds, urinary tract infections, and heart disease. Further studies are required to project the prospective benefits years down the road that we have confidently shown occur during the acute phase of NTSCI rehabilitation.

### 4.4. Nontraumatic Sci Specific Challenges

#### 4.4.1. Neurological Prognosis and Survival

Non-traumatic SCI is a large, overarching category of various diagnoses, and as such, the data on morbidity varies widely. In one retrospective cohort study of 174 participants that compared functional outcomes of traumatic spinal cord injury with non-traumatic spinal cord injury in Norway, they found that improvement in the American Spinal Injury Association Impairment Scale (AIS) did not differ between the two groups, though TSCI demonstrated significantly more urinary tract infections and NTSCI subjects were found to develop more pressure injuries; the length of stay in inpatient rehabilitation was on average 3.4 weeks longer in the traumatic SCI group [84]. However, a large retrospective cohort study in Israel noted that NTSCIs showed substantial neurologic recovery when evaluated based on Frankel grade as compared to their traumatic SCI counterparts during the first admission to rehabilitation. With variables such as age and gender controlled, the odds of recovery during rehabilitation were noted to be highest for benign tumors and disc herniation and lowest for multiple sclerosis [85]. Further studies are needed to explore the impact of traumatic versus non-traumatic etiology on spinal cord injury and its impact on functional outcomes. Regarding survival, data has shown that the oncologic status of non-traumatic SCI drives mortality. Mortality risk is more pronounced in individuals with nontraumatic SCIs from malignant neoplasms, with completeness of lesion having the largest impact on survival [86].

#### 4.4.2. Participation in Rehabilitation

Due to the heterogeneity of the non-traumatic spinal cord injury patient population, various factors play a role in the participation of patients in the inpatient rehabilitation setting. Patients with non-traumatic spinal cord injury often have varying ongoing treatments associated with their specific etiology, such as intravenous immunoglobin, immunosuppressive medications, and oncologic treatments. The requirement of additional therapies ideally would not hinder access to inpatient rehabilitation, but determination of feasibility is center-specific. Overall, there is evidence that individuals with nontraumatic spinal cord injuries do benefit from inpatient rehabilitation, with improved outcomes associated with admission to spinal cord injury-specific units compared to general rehabilitation units due to access to specialized services and education. However, there is a noted preference for the admission of traumatic SCI patients over those with non-traumatic etiology, despite evidence of improved outcomes [5].

## 5. Conclusions

Non-traumatic spinal cord injuries are heterogenous in etiology and presentation. Compressive and non-compressive etiologies often result in neurological deficit, decline in function, and pain in some cases. The heterogenicity of these conditions as well as the associated comorbidities often make the diagnosis challenging. Medical history and physical exam provide key diagnostic elements; however, due to difficulties differentiating within the various entities and the need for a close evaluation of the neuroanatomy, MRI is the goal standard for the diagnosis of most NTSCI. Associated pathology such as infection and oncological diagnosis increases the complexity level and often limits the participation and efficiency of the rehabilitation process. Nonetheless, comprehensive rehabilitation interventions are essential in improving the function, quality of life, and survival of patients with NTSCI.

## Figures and Tables

**Table 1:** Demographic, clinical, and neurological features of traumatic and nontraumatic SCI. Adopted with permission From the American Association of Neurological Surgeons (AANS); American Society of Neuroradiology (ASNR); Cardiovascular and Interventional Radiology Society of Europe (CIRSE); Cannadian International Radiology Assciation (CIRA); Congress of Neurological Surgeons (CNS); European Society of Minimally Invasive Neurological Therapy (ESMINT); European Society of Neuroradiology (ESNR); European Stroke Organization (ESO); Society for NeuroInterventional Surgery (SNIS); World Stroke Organization(WSO); et al. Multisociety Consensus Quality Improvement Revised Consensus Statement for Endovascular Therapy of Acute Ischemic Stroke. *Int. J. Stroke* **2018**, *13*, 612–632. [14] with permission from Elsevier.

	Total	T-SCI	NT-SCI	*p*-Value		Total	T-SCI	NT-SCI	*p*-Value
Sex				**(0.06)**	Clinical presentation				**(0.002)**
Male	75 (67%)	22 (81%)	53 (63%)		Tetraplegia	32 (29%)	14 (52%)	18 (21%)	
Female	37 (33%)	5 (19%)	32 (37%)		Paraplegia	80 (71%)	13 (48%)	67 (79%)	
	**Total**	**T-SCI**	**NT-SCI**	***p*-Value**		**Total**	**T-SCI**	**NT-SCI**	***p*-Value**
Age				**(0.0005)**	**Level of injury**				**(0.005)**
Range Mean ± SD	22–87 60 ± 14.8	22–77 52 ± 17.7	25–87 63 ± 12.6		Cervical Cervical and thoracic	30.5% 3.5%	44.5% 15.0%	26.0% -	
					Thoracic	57.0%	33.0%	65.0%	
					Lumbar-sacral	9.0%	7.5%	9.0%	
	**Total**	**T-SCI**	**NT-SCI**	***p*-Value**					
Completeness of injury				**(0.001)**					
Complete	8 (7%)	7 (21%)	2 (2%)						
Incomplete	104 (93%)	29 (79%)	83 (98%)						

**Table 2:** Intramedullary and extramedullary tumors and their locations.

	Extramedullary	
Intramedullary	Intradural	Extradural
Ependymoma	Meningioma	Metastasis
Astrocytoma	Malignant nerve sheet tumors	Vertebral primary & secondary tumors
Metastasis	Schwannomas	Chordoma
Arterial-venous malformation	Neurofibromas	Sarcomas
Hemangioblastoma	Lipoma	Lymphomas
	Leptomeningeal carcinomatosis	Plasmacytomas

**Table 3:** Clinical presentation of extramedullary versus intramedullary spinal cord tumors.

Extramedullary	Clinical Presentation	Intramedullary
Common	Local pain—vertebral pain	Rare
Common	Radicular pain	Rare
Less common	Funicular pain	Common
Yes, early	Upper motor neuron signs	Yes, late
Uncommon; if present segmental	Lower motor neuron signs	Prominent and diffuse
Ascending; sacral involvement	Paresthesia progression	Descending; sacral spearing, dissociate loss
Cauda equina (late)	Sphincter abnormalities	Conus lesions (early)

**Table 4:** Spine instability neoplastic scoring (SINS). Adopted from reference [54] Fisher, C.G.; DiPaola, C.P.; Ryken, T.C.; Bilsky, M.H.; Shaffrey, C.I.; Berven, S.H.; Harrop, J.S.; Fehlings, M.G.; Boriani, S.; Chou, D.; et al. A novel classification system for spinal instability in neoplastic disease: an evidence-based approach and expert consensus from the Spine Oncology Study Group. *Spine* **2010**, *35*, E1221–E1229.

Element	Score
**Location**	
Junctional (occiput-C2, C7-T2, T11-L1, L5-S1)	3
Mobile spine (C3-C6, L2-L4)	2
Semirigid spine (T3-T10)	1
Rigid spine (S2-S5)	0
**Pain with recumbency and/or movement of spine**	
Yes	3
Occasional, but not mechanical	1
No	0
**Bone lesion**	
Lytic	2
Mixed (lytic and blastic)	1
Blastic	0
**Radiographic spinal alignment**	
Subluxation or translation present	4
De novo deformity (kyphosis or scoliosis)	2
Normal alignment	0
**Vertebral body collapse**	
>50%	3
<50%	2
No collapse, with >50% of body involved	1
None	0
**Involvement of posterolateral spine elements (face, pedicle or costovertebral joint fracture or replacement with tumor)**	
Bilateral	3
Unilateral	1
None of the above	0
**Total score**	
Stable	0–6
Indeterminate	7–12
Unstable	13–18

**Table 5:** Functional Impairment Measure (FIM) and Length of Stay comparison; TSCI, Traumatic SCI; NTSCI, Non-Traumatic SCI; NR, Not Reported; NA Not Applicable.

Author (Country)	Years	Numbers (n) SCI Group		Age (Years) SCI Group		Admission FIM SCI Group		Discharge FIM SCI Group		Length of Stay (Days) SCI Group	
		TSCI	NTSCI	TSCI	NTSCI	TSCI	NTSCI	TSCI	NTSCI	TSCI	NTSCI
Mckinley et al. (USA) [79]	1992–99	86	86	NR	NR	36.7	37.0	68.0	55.8	41.35	22.38
Grazia Celani et al. (Italy) [80]	1989–99	642	217	34.3 ± 15.5	48.2 ± 18.1	NR	NR	NR	NR	143.1 ± 89.1	91.7 ± 78.9
Ostherthun et al. (Netherlands & Belgium) [83]	2002–07	389	530	43.4 ± 16.7	57.2 ± 14.5	NR	NR	NR	NR	227.6 ± 105.2	142.7 ± 110.5
New et al. (Australia) [4]	2002–06	1361	2241	46.0	67.0	38	53	74	76	44	21
New (Australia) [6]	1995–97	NA	70	NA	69	NA	37.5 ± 11.4	NA	52.0 ± 18.5	NA	55.8

## Data Availability

Data was obtained by reviewing published literature.
